# The Evaluation of Red Cell Distribution Width in Chronic Hemodialysis Patients

**DOI:** 10.1155/2014/754370

**Published:** 2014-03-30

**Authors:** Hikmet Tekce, Buket Kin Tekce, Gulali Aktas, Mehmet Tanrisev, Mustafa Sit

**Affiliations:** ^1^Department of Nephrology, Faculty of Medicine, Abant Izzet Baysal University, 14280 Bolu, Turkey; ^2^Department of Medical Biochemistry, Faculty of Medicine, Abant Izzet Baysal University, 14280 Bolu, Turkey; ^3^Department of Internal Medicine, Faculty of Medicine, Abant Izzet Baysal University, 14280 Bolu, Turkey; ^4^Department of Nephrology, Tepecik Education and Research Hospital, 35100 Izmir, Turkey; ^5^Department of General Surgery, Faculty of Medicine, Abant Izzet Baysal University, 14280 Bolu, Turkey

## Abstract

*Background*. Red cell distribution width (RDW) has been used as a marker of iron deficiency; however, it is accepted as a marker of cardiovascular survival. We aimed to study RDW levels in hemodialysis (HD) patients and the association between RDW and inflammatory, nutritional, and volume markers. *Methods*. We included 296 HD patients with sufficient iron storage and without anemia or hypervolemia. We grouped patients into four groups according to clinical parameters, albumin, and C-reactive protein (CRP). *Results*. The lowest RDW levels were found in group 1 (13.2%). Although RDW of group 2 was higher than that of group 1, it was still in normal range (14.7% versus 13.2%, *P* = 0.028). RDW levels of groups 3 (17.8%) and 4 (18.5%) were significantly higher than those of groups 1 and 2 and above normal range. A positive correlation was detected between RDW and HD duration, interdialytic weight gain (IDWG), serum phosphate, and CRP levels and a negative correlation was detected with serum albumin. HD duration, CRP, IDWG, and serum albumin have been found as independent predictors of RDW elevation. *Conclusions*. Results of the present study reflect adverse effects of inflammation, malnutrition, and excess IDWG on RDW elevation in an HD study cohort with sufficient iron storage and without anemia and hypervolemia.

## 1. Introduction

Red cell distribution width (RDW) is a quantitative marker of the variability in size of erythrocytes [1]. It is a routine assay of complete blood count tests and does not require an additional cost [2]. Elevated RDW reflects increased size variations of red blood cells which indicates altered erythrocyte life span or dysfunctional erythrocytes [2, 3]. RDW is usually used for differential diagnosis of anemia, especially as a marker in iron deficiency anemia [4]. In addition, RDW has been found as a predictor of mortality in the general population [5] and in several conditions including: acute and chronic heart failure [6, 7], acute pulmonary embolism [8], myocardial infarction [9], peripheral arterial disease [10], acute renal failure required renal replacement therapy [11], and kidney transplant recipients [12].

Although RDW has been associated with survival in acute and chronic diseases, the underlying physiopathological mechanism remains unclear. Recent studies investigated pathophysiological mechanisms of negative cardiovascular outcomes in these populations. Authors hypothesized that the size variations of erythrocytes reflected the functional iron status and functions of bone marrow [3]. Furthermore, endothelial dysfunction [13], microalbuminuria, which is a marker of cardiovascular risk [14], inflammation [15], and increased oxidative stress [16] have been suggested as responsible of increased mortality. However, these mechanisms are still controversial. Besides, there is no data in the literature about RDW levels in end stage renal disease (ESRD) patients independent of anemia and volume status and its association with clinical parameters. Therefore, we aimed to study RDW levels in hemodialysis (HD) patients without anemia and with sufficient iron storage and also studied the association between RDW and inflammatory, nutritional and volume markers.

## 2. Materials and Methods

We retrospectively analyzed 514 patients who received HD treatment between 2008 and 2012. We included into the study stable chronic HD patients with sufficient iron storage and without anemia or hypervolemia. All patients included in the study were receiving HD treatment for four-five hours three times a week. Patients in the study used polysulfone membranes and bicarbonate dialysates. Blood flow rate was 350–500 mL/min for all study participants. We recorded age, gender, and HD duration, underlying cause of ESRD, predialysis systolic and diastolic blood pressures, previous renal transplantation history, presence of diabetes mellitus (DM) or coronary artery disease (CAD), interdialytic weight gain (IDWG), and treatment status (iron, erythropoietin, or antiphosphate) of the study population. Laboratory parameters such as hemoglobin (Hb), hematocrit, RDW, mean corpuscular volume (MCV), mean cell hemoglobin, mean corpuscular hemoglobin concentration, white blood cell count, platelet count, blood urea nitrogen, creatinine, glucose, electrolyte levels (Na, K, Ca, and P), intact parathyroid hormone (iPTH), total cholesterol (T-chol), high-density lipoprotein cholesterol (HDL-chol), low-density lipoprotein cholesterol (LDL-chol), triglyceride, albumin, C-reactive protein (CRP), ferritin, transferrin saturation, urea reduction ratio (URR), kT/V, and cardiothoracic index obtained in the predialysis period.

Exclusion criteria were as follows: hemoglobin <12 g/dL, hematocrit < 36%, ferritin < 500 mg/dL, transferrin saturation <20%, receiving erythropoietin or iron preparations, insufficient HD (kT/V < 1.4 and/or URR < 0.65), active infection or inflammation in clinical records, and subjects older than 70 years of age. We also did not include patients with suspected hypervolemia (IDWG > 2.5 kg, cardiothoracic index > 50%, recorded peripheral edema or cardiovascular loading signs, and predialysis arterial blood pressure > 140/90 mm Hg). We recorded data of 514 patients initially but 218 were excluded from the study according to exclusion criteria.

The etiologies of the ESRD in our study population were as follows: DM (*n* = 91), hypertension (*n* = 64), glomerulonephritis (*n* = 38), urolithiasis (*n* = 23), polycystic kidney disease (*n* = 21), tubulointerstitial disease (*n* = 17), vesicoureteral reflux/pyelonephritis (*n* = 11), and unknown (*n* = 31). We grouped the remaining 296 patients into 4 groups according to clinical parameters, albumin, and C-reactive protein (CRP): group 1 (*n* = 92), no malnutrition or inflammation (albumin > 3.5 g/dL and CRP < 5 mg/L); group 2 (*n* = 81), inflammation alone (albumin > 3.5 g/dL and CRP > 5 mg/L); group 3 (*n* = 74) malnutrition alone (albumin < 3.5 g/dL and CRP < 5 mg/L); and group 4 (*n* = 49) both malnutrition and inflammation (albumin < 3.5 g/dL and CRP > 5 mg/L).

### 2.1. Statistical Analysis

SPSS software version 15.0 (SPSS; Chicago, IL, USA) is used for statistical analysis. Continuous variables were presented as mean ± standard deviation while categorical variables were presented as the percentage. The normal distribution of all variables was tested using the Shapiro-Wilk test. Pearson's and Spearman correlation tests are used to determine the correlation between variables. ANOVA test was applied to compare continuous variables, and the difference between subgroups was assessed with the post hoc Tukey's test. *χ* ^2^ test was used to compare for categorical data. We compared nonparametric data with Kruskal-Wallis test. A value of *P* < 0.05 was considered statistically significant. A multiple linear regression model was used to identify independent predictors of elevated RDW. The model fit was assessed using appropriate residual and goodness-of-fit statistics. A 5% type-I error level was used to infer statistical significance.

## 3. Results

Clinical and demographic data of 296 patients are summarized in Table 1. There was no difference between study groups in terms of age, gender, and HD durations. On the other hand, presence of DM and CAD and antiphosphate drug usage was significantly different between study groups.

**Figure 1:**
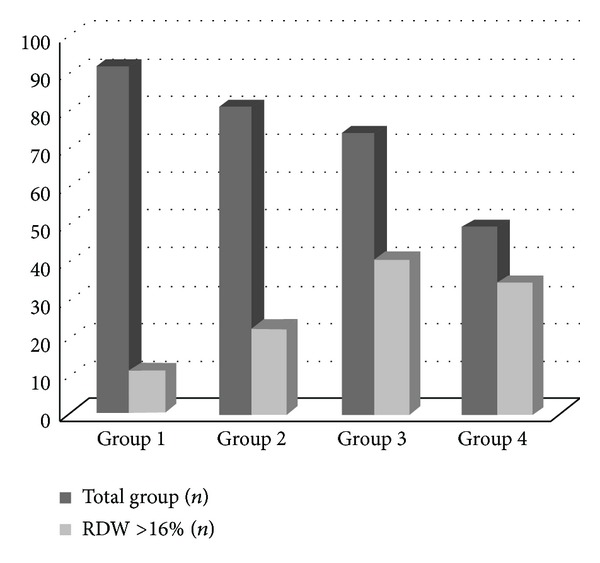
Distribution of patients with elevated red cell distribution width (RDW) in study groups.

The clinical and laboratory characteristics of four study groups were shown in Table 2. There was no significant difference between study groups in terms of laboratory parameters except RDW, creatinine, T-chol, LDL-chol, triglyceride, albumin, and CRP. Mean RDW of all study population was 17.2 ± 1.9% (reference range: 12–16%). The lowest RDW levels in study cohort were found in group 1, even within normal range (13.2 ± 1.4%). Although RDW of group 2 was higher than group 1, it was still in normal range (14.7 ± 1.6% versus 13.2 ± 1.4%,* post hoc P* = 0.028). RDW levels of groups 3 (17.8 ± 1.9%) and 4 (18.5 ± 2.0%) are significantly higher than groups 1 (*post hoc P* = 0.017, *P* = 0.004, resp.) and 2 (*post hoc P* = 0.031, *P* = 0.019, resp.) and above normal range. RDW levels were not significantly different in patients with and without hepatitis B and hepatitis C (16.7%, 16.2%, and 17.1%, *P* > 0.05, resp.). The numbers of patients with high RDW for each study groups have been described in Figure 1.

Table 3 shows bivariate correlation analyses of RDW levels and clinical and laboratory parameters of study population. There was a positive correlation between RDW and HD duration (*r* = 0.251, *P* = 0.048), interdialytic weight gain (*r* = 0.395, *P* = 0.021), serum phosphate (*r* = 0.296, *P* = 0.038), and CRP (*r* = 0.415, *P* = 0.014) levels and a negative correlation with serum albumin (*r* = −0.602, *P* = 0.006). There was no bivariate correlation between RDW and other study parameters.

The results of multivariate (backward) analyses were listed in Table 4 indicating risk factors for high RDW. HD duration, CRP, and interdialytic weight gain were independent positive predictors of RDW elevation, while serum albumin was a negative predictor in multivariate analysis. The other parameters (age, gender, presence of diabetes mellitus or coronary artery disease, mean arterial pressure, hemoglobin, ferritin, transferrin saturation, phosphate, parathyroid hormone, and kT/V) in multivariate analyses were not significantly associated with RDW.

## 4. Discussion

The results of the present study conducted in an HD population with sufficient iron stores and without anemia and hypervolemia indicates the following: (1) RDW is increased above normal reference range in ESRD patients, especially in the subgroup of patients with inflammation and malnutrition, (2) independent risk factors associated with RDW elevation were HD duration, CRP, interdialytic weight gain, and albumin levels, (3) RDW was positively correlated with HD duration, interdialytic weight gain, serum phosphate and CRP levels, and negatively correlated with serum albumin.

There are several reports in the literature described RDW changes in patients with impaired renal functions. Docci et al. described RDW changes in chronic kidney disease patients for the first time in a preliminary study [17]. In their study, RDW has been found to be increased in chronic HD patients compared to healthy subjects. Data in the literature have suggested the association between RDW and renal functions subsequently. Lippi et al. showed negative and gradual relation between RDW and renal functions in their study group of congestive heart failure patients [18]. They concluded that decreased GFR predicted elevated RDW independent of age, gender, MCV, and hemoglobin. Our results indicating increased RDW in overall study population suggest the results of previous studies.

Is high RDW in renal dysfunction associated with survival? A recent study showed that RDW was independently and closely associated with mortality in patients with acute renal injury in intensive care unit [11]. Similarly, high RDW has been found to be related with mortality in kidney transplant recipients [12]. A prospective longitudinal study revealed that high RDW was an independent predictor of mortality from all causes in patients with chronic HD patients [19]. However, there is little data in the literature studied the underlying causes of the association between high RDW and mortality in patients with renal impairment. The results of present study point a deterioration in inflammatory, nutritional, and volume status independent of anemia for possible causes of worse outcome. Solak et al.'s study was important, which indicated the association between increased RDW and endothelial dysfunction [13]. They figured out an association independent of anemia and inflammation between RDW and endothelial dysfunction and carotid intima media thickness which was calculated by flow-mediated dilatation. A study in literature showed independent and strong relation between RDW and hsCRP and erythrocyte sedimentation rate in 3845 healthy subjects [20]. Authors in another study found that RDW was strongly associated with CRP in 1489 CAD patients; moreover, these two markers were found to be independent predictors of mortality of all causes after 8.4–15.2 years of followup [15]. In contrast, there are also several studies which could not demonstrate an association between inflammation and RDW [21]. RDW has been found to be related with inflammatory and nutritional markers and a predictor of mortality in a study with 195 systolic heart failure patients [22]. To our knowledge, no data studied the association between malnutrition and RDW in HD patients. Therefore, the results of the present study are important, indicating significant association not only between RDW and CRP but also between RDW and hypoalbuminemia, which is a predictor of malnutrition and mortality. These findings may reflect negative effects of inflammation and malnutrition on the association between mortality and elevated RDW in ESRD patients.

Naturally, this work has some limitations because of its retrospective design. First of all, retrospective design of the study makes it difficult to understand clearly the cause-and-effect relationship. Second, our results may not be proper to be generalized because of the single center character of our work. Third, RDW measurement was performed based on a single value and cannot consider the relationship between possible timely changes and clinical parameters. However, great sample size and low missing data rates strengthen our results. It is also important that these results reflect an HD cohort after adjusting anemia and hypervolemia because it is difficult to obtain such a population this big size, prospectively.

## 5. Conclusions

The results of the present study reflect negative effects of inflammation, malnutrition, and interdialytic excess weight gain on RDW elevation in an HD study cohort with sufficient iron storage and without anemia and hypervolemia. However, prospective, multicenter studies are needed to observe possible other pathophysiological mechanisms of RDW elevation in ESRD patients.

## Figures and Tables

**Table 1:** The clinical and demographic characteristics of the patients.

Parameter	Group 1 (*n* = 92)	Group 2 (*n* = 81)	Group 3 (*n* = 74)	Group 4 (*n* = 49)	*P*
Age (years)	67.6 ± 11.3	63.5 ± 9.2	70.7 ± 14.9	64.9 ± 10.8	>0.05*
Gender (female/male) (*n*)	39/53	37/44	30/44	21/28	>0.05^‡^
HD duration (months)	66.1 ± 24.9	62.8 ± 20.3	68.7 ± 31.3	71.2 ± 26.3	>0.05^†^
Presence of DM (yes/no) (*n*)	**37/55**	**29/52**	**23/51**	**19/30**	0.029^‡^
Presence of CAD (yes/no) (*n*)	**17/75**	**16/65**	**12/62**	**13/36**	0.043^‡^
Presence of Rtx history (yes/no) (*n*)	4/88	2/79	3/71	1/48	>0.05^‡^
Predialysis systolic BP (mm Hg)	118.9 ± 15.7	115.9 ± 18.1	110.3 ± 14.1	112.7 ± 12.9	>0.05*
Predialysis diastolic BP (mm Hg)	74.2 ± 12.3	71.8 ± 13.4	67.5 ± 10.1	69.2 ± 11.6	>0.05*
Iron/EPO treatment (present/absent)	0/296	0/81	0/74	0/49	—
Anti-P treatment (present/absent) (*n*)	**55/37**	**38/43**	**21/53**	**19/30**	0.013^‡^
Interdialytic weight gain (kg)	1.82 ± 0.52	1.69 ± 0.47	1.95 ± 0.44	1.91 ± 0.54	>0.05*
Cardiothoracic index (%)	39.1 ± 7.7	41.2 ± 6.4	38.2 ± 7.6	43.5 ± 5.1	>0.05*

Differences assessed by ANOVA* (for parametrical data) or Kruskal-Wallis test^†^ (for nonparametric data) for numerical variables and by Chi-square test^‡^ for categorical variables. Statistically significance: *P* < 0.05.

HD: hemodialysis, DM: diabetes mellitus, CAD: coronary artery disease, Rtx: renal transplantation, BP: blood pressure, EPO: erythropoietin, and P: phosphate.

**Table 2:** The comparative clinical and laboratory data of HD patients.

Parameter	Group 1 (*n* = 92)	Group 2 (*n* = 81)	Group 3 (*n* = 74)	Group 4 (*n* = 49)	*P*
Hemoglobin (g/dL)	12.9 ± 0.8	13.1 ± 1.0	13.0 ± 1.0	12.8 ± 0.7	>0.05*
Hematocrit (%)	41.8 ± 4.6	43.7 ± 5.8	40.2 ± 4.2	39.7 ± 4.1	>0.05*
RDW (%)	13.2 ± 1.4	14.7 ± 1.6^‡^	17.8 ± 1.9^§^	18.5 ± 2.0^¶^	0.014*
MCV (fL)	85.9 ± 7.4	83.7 ± 6.1	88.5 ± 5.2	91.4 ± 6.0	>0.05*
MCH (pg)	28.4 ± 2.7	27.9 ± 3.0	31.3 ± 2.9	30.2 ± 3.5	>0.05*
MCHC (g/dL)	34.5 ± 1.4	35.2 ± 1.7	34.0 ± 1.3	33.1 ± 1.0	>0.05*
TS (%)	33.8 ± 17.1	36.1 ± 14.7	34.5 ± 19.8	38.6 ± 18.4	>0.05^†^
Ferritin (ng/mL)	627.1 ± 112.6	734.6 ± 140.3	662.8 ± 130.8	704.4 ± 142.9	>0.05*
Creatinine (mg/dL)	8.9 ± 3.1	10.5 ± 3.9	8.2 ± 4.0	9.6 ± 4.8	0.026^†^
Calcium (mg/dL)	9.1 ± 1.6	9.6 ± 1.8	8.9 ± 1.3	9.2 ± 1.6	>0.05*
Phosphate (mg/dL)	5.1 ± 1.0	5.3 ± 1.1	4.5 ± 0.8	4.7 ± 0.9	>0.05*
Total cholesterol (mg/dL)	178.8 ± 60.1	193.7 ± 81.5	162.4 ± 65.8	155.7 ± 58.1	0.045^†^
Triglyceride (mg/dL)	183.2 ± 76.4	191.3 ± 62.8	154.4 ± 58.7	159.2 ± 65.5	0.038^†^
HDL-cholesterol (mg/dL)	32.1 ± 7.4	37.2 ± 8.5	33.8 ± 6.8	30.7 ± 6.2	>0.05*
LDL-cholesterol (mg/dL)	122.3 ± 39.7	145.7 ± 46.8	110.5 ± 37.2	103.4 ± 48.7	0.029^†^
Albumin (g/dL)	4.1 ± 0.4	3.8 ± 0.3	3.3 ± 0.3	3.0 ± 0.2	0.035*
CRP (mg/L)	3.5 ± 2.4	7.9 ± 2.1	4.1 ± 2.7	9.6 ± 4.2	0.024*
iPTH (pg/mL)	286.5 ± 132.7	261.3 ± 102.2	278.4 ± 130.1	301.2 ± 119.7	>0.05^†^
URR (%)	0.71 ± 0.05	0.74 ± 0.06	0.69 ± 0.03	0.70 ± 0.04	>0.05*
kT/V	1.54 ± 0.21	1.50 ± 0.26	1.48 ± 0.31	1.53 ± 0.25	>0.05*

*One-way ANOVA (for parametric data).

^†^Kruskal Wallis test (for nonparametric data).

^‡^RDW difference between group 1 and group 2, post hoc *P* = 0.028 (Tukey's test).

^§^RDW difference between group 3 and group 1, post hoc *P* = 0.017; group 3 versus group 2, post hoc *P* = 0.031 (Tukey's test).

^¶^RDW difference between group 4 and group 1, post hoc *P* = 0.004; group 4 versus group 2, post hoc *P* = 0.019; group 4 versus group 3, post hoc *P* = 0.041 (Tukey's test).

RDW: red cell distribution width, MCV: mean corpuscular volume, MCH: mean cell hemoglobin, MCHC: mean corpuscular hemoglobin concentration, TS: transferrin saturation, LDL: low-density lipoprotein cholesterol, HDL: high-density lipoprotein, CRP: C-reactive protein, iPTH: intact parathyroid hormone, and URR: urea reduction ratio.

**Table 3:** Bivariate correlation analyses of RDW levels and various clinical and laboratory parameters of study population.

Parameter	*r*	*P*
Age	0.134	0.463*
Gender	0.078	0.822^†^
HD duration	**0.251**	0.048^†^
Presence of DM	0.161	0.351^†^
Presence of CAD	0.102	0.125^†^
Mean arterial pressure	0.086	0.301*
Interdialytic weight gain	**0.395**	0.021*
Cardiothoracic index	0.188	0.091*
Hemoglobin	−0.077	0.224*
Serum ferritin	−0.180	0.098*
Transferrin saturation	−0.134	0.136^†^
Serum creatinine	0.104	0.177^†^
Serum calcium	−0.062	0.455*
Serum phosphate	**0.296**	0.038*
iPTH	0.077	0.502^†^
Serum total cholesterol	0.128	0.211^†^
Serum triglyceride	0.055	0.401^†^
Serum albumin	**−0.602**	0.006*
CRP	**0.415 **	0.014*
URR	−0.115	0.102*
kT/V	−0.162	0.088*

*Pearson correlation test.

^†^Spearman's correlation test.

HD: hemodialysis, DM: diabetes mellitus, CAD: coronary artery disease, iPTH: intact parathyroid hormone, CRP: C-reactive protein, and URR: urea reduction ratio.

**Table 4:** Backward linear regression analyses of factors related to elevated RDW.

	Beta	*t*	*P*
Hemodialysis duration	0.171	1.961	0.043
C-reactive protein	0.507	7.912	0.011
Interdialytic weight gain	0.146	2.337	0.027
Serum albumin	−0.638	−9.134	0.003
